# Rapid MRI Abdomen for Assessment of Clinically Suspected Acute Appendicitis in the General Adult Population: a Systematic Review

**DOI:** 10.1007/s11605-023-05626-8

**Published:** 2023-04-20

**Authors:** Dongchan Kim, Benjamin Luke Woodham, Kathryn Chen, Vinushan Kuganathan, Michael Benjamin Edye

**Affiliations:** 1 School of Medicine, Western Sydney University, Campbelltown, N.S.W. Australia; 2 Department of General Surgery, Blacktown and Mount Druitt Hospitals, Blacktown Road, Blacktown, N.S.W. Australia

**Keywords:** MRI, Magnetic resonance imaging, Appendicitis, Appendicectomy, Appendix, Systematic review

## Abstract

**Objectives:**

To perform a systematic review on the use of magnetic resonance imaging (MRI) of the abdomen to evaluate clinically suspected appendicitis in the general adult population. We examined the diagnostic accuracy, the reported trends of MRI use, and the factors that affect the utility of MRI abdomen, including study duration and cost-benefits.

**Methods:**

We conducted a systematic literature search on PubMed, MEDLINE, Embase, Web of Science, and Cochrane Library databases. We enrolled primary studies investigating the use of MRI in diagnosing appendicitis in the general adult population, excluding studies that predominantly reported on populations not representative of typical adult appendicitis presentations, such as those focusing on paediatric or pregnant populations.

**Results:**

Twenty-seven eligible primary studies and 6 secondary studies were included, totaling 2,044 patients from eight countries. The sensitivity and specificity of MRI for diagnosing appendicitis were 96% (95% CI: 93–97%) and 93% (95% CI: 80–98%), respectively. MRI can identify complicated appendicitis and accurately propose alternative diagnoses. The duration of MRI protocols in each primary study ranged between 2.26 and 30 minutes, and only one study used intravenous contrast agents in addition to the non-contrast sequences. Decision analysis suggests significant benefits for replacing computed tomography (CT) with MRI and a potential for cost reduction. Reported trends in MRI usage showed minimal utilisation in diagnostic settings even when MRI was available.

**Conclusions:**

MRI accurately diagnoses appendicitis in the general adult population and improves the identification of complicated appendicitis or alternative diagnoses compared to other modalities using a single, rapid investigation.

## Introduction

General surgeons encounter clinically suspected appendicitis most commonly in adults,^1^ with an estimated 87% of appendicitis cases occurring in individuals aged over 15.^2^ Surgeons reviewing patients suspected of acute appendicitis often utilise imaging to enhance diagnostic certainty and prevent unnecessary invasive procedures.^3^ The rate of imaging use and the choice of modality varies according to local practice.^4–7^

Traditionally, imaging has been sparingly used for suspected appendicitis in the UK and Australia.^4,5^ In one Australian report, only 25% of patients received imaging, with this figure being mostly ultrasound.^8^ Although reliance on the clinical acumen of surgeons can help avoid the issues surrounding the routine use of CT scans, minimal use of imaging results in an increased length of stay^5^ and a higher negative appendicectomy rate.^9^ Clinical scoring systems can improve outcomes such as length of stay,^10^ but still result in a negative appendicectomy rate of 10%.^11^

In some countries, such as the USA or the Netherlands, imaging is routinely employed for assessing possible appendicitis cases in the general adult population with imaging rates reported at 99%.^6,7^ Computed tomography (CT) is a popular modality for diagnosing acute appendicitis, due to its high sensitivity (95%; 95% CI: 93–96%) and specificity (94%; 95% CI: 92–95%).^12^ However, ionising radiation from CT scans increases the cumulative risk of carcinogenesis in patients.^13,14^ The incidence rate ratio of leukaemia and myelodysplasia for patients that have undergone CT of the abdomen and pelvis is 3.24 (95% CI: 2.17–4.84).^15^ Furthermore, the incidence of contrast-induced nephropathy nears 25% in patients with pre-existing renal impairments.^16^ Intravenous contrast is a common allergic and anaphylactic reaction trigger, and can also cause fluid extravasation and hazardous interactions with common medications such as metformin.^17^ Ultrasound is an alternative, radiation-free imaging modality also commonly employed to detect acute appendicitis, with superior safety when compared to CT.^18,19^ However, ultrasound is limited by a low sensitivity (69%; 95% CI: 59–78%) and specificity (81%; 95% CI: 73–88%) when evaluating suspected appendicitis, with inconclusive results in nearly 48% of cases.^20,21^

A US study reported a system-wide trend towards significantly increased use of CT in emergency departments (ED) for assessing patients with abdominal pain, without a corresponding increase in detecting surgical emergencies.^22^ Clinicians and patients are understandably keen to reduce the number of missed appendicitis cases and improve the diagnosis of other conditions. However, the increasing use of CT inevitably increases patient exposure to ionising radiation and intravenous contrast agents. An ideal alternative imaging modality would be affordable and rapid with high sensitivity and specificity for all common causes of abdominal pain whilst not exposing patients to ionising radiation and contrast agents.

Magnetic resonance imaging (MRI) has been widely used as an alternative imaging modality for diagnosing acute appendicitis in paediatric or pregnant patients where avoiding ionising radiation is a priority.^23,24^ The benefit of avoiding radiation exposure and contrast agents can be extended to the general adult population, although the relatively high cost and limited availability of MRI have historically remained as impediments to widespread use for these purposes.^25,26^

A 2021 Cochrane Review reported that rapid MRI abdomen (with a total study time lasting 30 minutes or less) may entail numerous additional advantages that may help overcome its perceived high cost, including a low false-positive rate and a low negative appendicectomy rate.^26^ Appendicitis is a high incidence condition, estimated at 100 (95% CI: 91–110) per 100,000 person-years,^27^ so substituting contrast-enhanced CT with MRI in this patient population has the potential for significant system-wide improvements in patient safety and outcomes.

Existing literature about MRI use for suspected appendicitis often includes high proportions of pregnant and paediatric patients, well above their proportions of the overall population, which is understandable given that these populations have led the way in the use of MRI. The anatomical and physiological differences between demographic groups may cause significant bias if the results from those subgroups are generalised to the adult population. The assessment of the utility of abdominal MRI outside these groups requires a dedicated study of the general (non-pregnant) adult population. The 2021 Cochrane Review examined the diagnostic accuracy of MRI for appendicitis^26^ in a population including large numbers of pregnant and paediatric patients, as well as reporting on the general adult subgroup. However, the scope of this meta-analysis was limited to reporting the pooled sensitivity and specificity of MRI, and two additional primary studies have been published since. We aimed to provide an updated systematic review on the use of MRI to assess clinically suspected appendicitis in the general adult population by reviewing the diagnostic accuracy and other knowledge gaps, such as the ability of MRI to identify complicated appendicitis, to identify alternative diagnoses, the reported trends of use, time requirements, cost-benefits, and potential impact on decision analyses.

## Method

We conducted our literature search in accordance with the Preferred Reporting Items for Systematic Reviews and Meta-Analyses guidelines^28^ using PubMed, Ovid MEDLINE, Ovid Embase + Embase Classic, Web of Science, and Cochrane Library with the search keywords “magnetic resonance imaging”, “MRI”, “appendicitis”, “appendix”, “appendicectomy”, and “appendectomy”. The studies were compiled and merged using the Zotero reference management software, and then duplicates, retractions, and studies lacking abstracts were removed. The remaining articles were screened for relevance to the use of MRI for detecting appendicitis.

After the initial screening, we excluded articles that met the exclusion criteria, formulated a priori. Studies were excluded if full texts could not be found, if they were case reports (1–2 patients), if they lacked explicit patient inclusion and exclusion criteria, or if they were written in languages other than English. Review articles, opinion pieces, book reviews, and study protocols were also excluded. Secondary studies were not excluded and their bibliographies were reviewed as an ancestral search to identify any further primary studies.

Two authors reviewed the remaining list of potentially eligible studies to determine which studies satisfied the inclusion criteria, formulated a priori. Studies were included if they reported the results of MRI scans evaluating suspected appendicitis cases in populations predominated by non-pregnant adults. Studies that reported only on pregnant or paediatric patients were excluded by this method.

The following information was then extracted from each study: name of the first author, year published, national location, study type, study duration, study setting, reference standard, the total number of patients, number of patients that underwent appendicectomy, prevalence of appendicitis, proportion of women, mean age, age range, number of subgroup patients, true positives, false positives, false negatives, true negatives, sensitivity, specificity, number of histologically proven appendicitis, number of alternative diagnoses from MRI results, number of cases with alternative diagnosis as final diagnosis, MRI sequence, and MRI scanning time. If available or possible, the positive predictive value (PPV) and the negative predictive value (NPV) of reported MRI features for discrimination of complicated from simple appendicitis were extracted or calculated.

The Quality Assessment of Diagnostic Accuracy Studies 2 (QUADAS-2) was employed to assess the validity and applicability of the included studies reporting on diagnostic outcomes.^29^

## Results

**Fig. 1:**
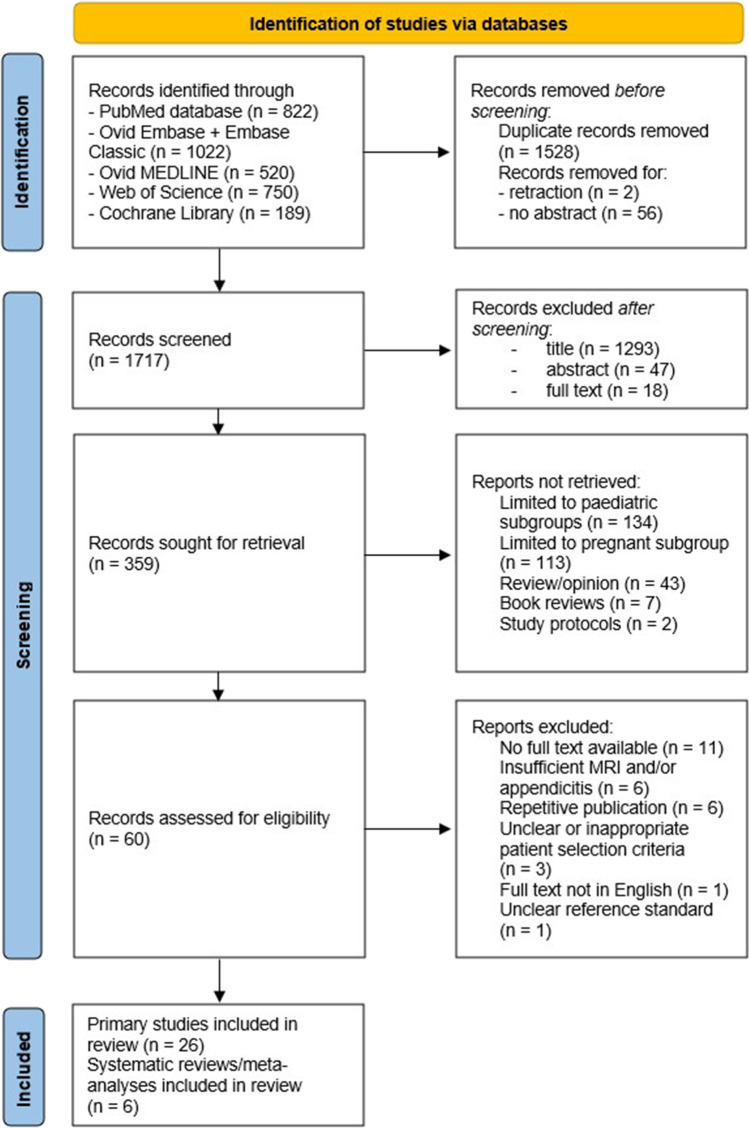
PRISMA flowchart detailing the literature search results

**Fig. 2:**
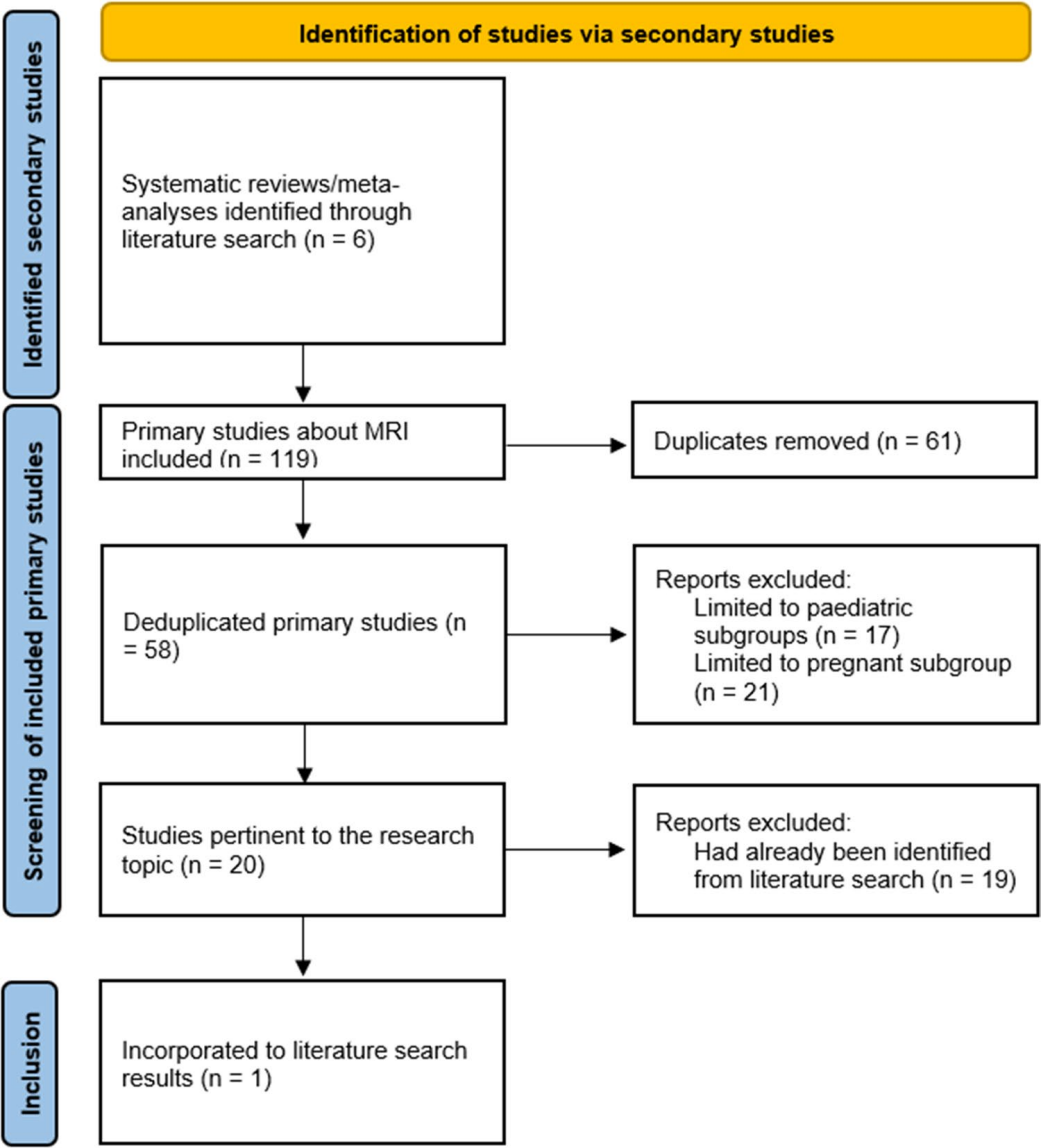
PRISMA flowchart detailing the ancestral search from bibliographies of secondary studies identified

The literature search identified a total of 3,303 studies from across PubMed, EMBASE, MEDLINE, Web of Science, and Cochrane Library databases. Twenty-six primary studies and six secondary studies were selected, as shown in the PRISMA flowchart in Fig. 1. One new primary study was identified in the ancestral searching of the bibliographies of the identified secondary studies, as shown in Fig. 2. In total, there were 33 studies identified by our systematic review.^5,26,30–60^

There were no randomised controlled trials (RCT) identified. Of the 33 studies identified in our systematic review, there were 16 primary cohort studies that examined the diagnostic accuracy of MRI for appendicitis with a total of 2,044 patients.^30–46^ Table 1 summarises the characteristics of these 16 studies, including their study designs, demographic information, and reference standards. All 16 studies employed non-contrast MRI sequences and only one study added a gadolinium contrast sequence to their multiple non-contrast sequences.^43^ Table 2 shows the MRI characteristics and diagnostic outcomes, which also shows that all the published MRI protocols required no more than 30 minutes for all sequences and that half the published MRI protocols required no more than 15 minutes.

**Table 1:** Characteristics of the included primary studies identified in the systematic review that reported on diagnostic outcomes

First author		Year		Location		Study type		Study duration (months)		Total patients		Mean age (range) (years)		Proportion of women		Number of operative patients		Reference Standard	Reference standard positive appendicitis	
S Serinsoz^30^		2021		Turkey		Retrospective, centre unknown		24		70		32 (11–71)		63%		40		Surgical results; unclear for non-operated patients	37/70 (53%)	
G Islam^31^		2021		India		Prospective, single-centre		18		67		24 (6–70)		37%		39		Histopath; or clinical follow-up	45/67 (67%)	
A Inoue^32^		2019		Japan		Retrospective, single-centre		10		51		35 (6–90)		53%		25		Surgery; or clinical follow-up and CT	35/51 (69%)	
M Repplinger^33^		2018		USA		Prospective, single-centre		30		198		32 (12–81)		58%		Not reported		Expert panel; Histopath or clinical follow-up	64/198 (32%)	
S Byott^34^		2016		England		Prospective, single-centre		60		468		Median 27 (7–59)		74%		116		Surgical, histological, and clinical follow-up	38/468 (8%)	
I Petkovska^35^		2016		USA		Retrospective, single-centre		24		253		Not reported		Not reported		Not reported		Expert panel; surgical results; or clinical follow-up	35/253 (14%)	
H Gielkens^36^		2016		Netherlands		Prospective, single-centre		18		112		Median 22 (12–55)		100%		51		Histopath; or clinical follow-up	29/112 (26%)	
M Leeuwenburgh^37^		2014		Netherlands		Prospective, multi-centre		6		223		35 (18–84)		62%		128		Expert panel; surgical findings, histopath, and clinical-follow-up	117/223 (52%)	
S Avcu^38^		2013		Turkey		Prospective, single-centre		12		55		36 (17–83)		43%		40		Histopath; unclear for non-operated patients	40/55 (73%)	
First Author	Year		Location		Study type		Study duration (months)		Total patients		Mean age (range) (years)		Proportion of women		Number of operative patients		Reference Standard			Reference standard positive appendicitis
B Zhu^39^	2012		China		Prospective, single-centre		24		41		42 (range not reported)		56%		41		Surgery/histopath			36/41 (88%)
E Inci^40^	2011		Turkey		Prospective, single-centre		11		119		27 (17–72)		36%		92		Histopath; unclear for non-operated patients			79/119 (66%)
E Chabanova^41^	2011		Denmark		Prospective, single-centre		Not reported		48		37 (18–70)		60%		48		Surgery/histopath			30/48 (63%)
L Cobben^42^	2009		Netherlands		Prospective, single-centre		22		138		Not reported (6–80)		58%		64		Histopath; or clinical follow-up			62/138 (44.9%)
A Singh^43^	2008		USA		Retrospective, multi-centre		72		67		34 (11–69)		91%		Not reported		Surgical results; unclear for non-operated patients			12/67 (18%)
N Nitta^44^	2005		Japan		Prospective, single-centre		Not reported		37		37 (16–69)		51%		31		Surgery/histopath; clinical follow-up			29/37 (43%)
L Incesu^46^	1997		Turkey		Prospective, single-centre		22		60		20 (14–71)		55%		39		Surgery/histopath; or clinical follow-up			34/60 (57%)

**Table 2:** MRI Characteristics and diagnostic outcomes reported by included primary studies

First author	MRI Sequences	MRI duration (min)	Number of histopatho-logically proven appendicitis	Number of cases of alternative diagnoses from pre-treatment MRI	Number of cases of alternative diagnoses after definitive treatment	TP	FP	FN	TN	Sensitivity (%)	Specificity (%)
S Serinsoz^30^	DWI ssEPI + FS, T2 TSE, T2 STIR/TIRM	7	37 (52.9%)	Not reported	Not reported	37	3	0	30	100	90.9
G Islam^31^	T2 and DWI	14	34 (50.7%)	6 (9.0%)	6 (9.0%)	42	3	3	19	93.3	86.4
A Inoue^32^	T2 HASTE and DWI	Not reported	25 (49.0%)	Not reported	16 (31.4%)	31	6	4	10	88.6	62.5
M Repplinger (J Harringa)^33^	T2-SS FSE, T1 3D spoiled GRE, DWI	30	Not reported	51 (25.8%)	54 (27.3%)	62	14	2	120	96.9	89.6
S Byott^34^ ^a^	HASTE	15	36 (7.7%)	82 (17.5%)	82 (17.5%)	34	3	3	428	91.9	99.3
I Petkovska^35^ ^b^	multiplanar single-shot T2 ± SPAIR FS	14 (mean)	Not reported	Not reported	Not reported	34	1	1	217	97.1	99.5
H Gielkens^36^ ^c^	T2 TSE, T1 GRE	22	29 (28.2%)	22 (21.4%)	Not reported	25	0	3	75	89.3	100
M Leeuwenburgh^37^	T2 FSE ± FS, DWI	15	114 (51.1%)	27 (12.1%)	Not reported	113	7	4	99	96.6	93.4
S Avcu^38^	DWI, bSSFP, STIR	2.26	40 (72.7%)	0 (0%)	0 (0%)	39	0	1	15	97.5	100
B Zhu^39^	T2 FSE, bSSFP + FS	2.7	36 (87.8%)	5 (12.2%)	5 (12.2%)	33	0	3	5	91.7	100
E Inci^40^	T1 FSE, T2 FSE ± FS, DWI	4	79 (66.4%)	Not reported	7 (5.9%)	78	0	1	40	98.7	100
E Chabanova^41^ ^d^	T1 SE, T2 FSE, STIR	20	30 (62.5%)	15 (31.3%)	14 (29.2%)	25	3	5	15	83.3	83.3
L Cobben^42^	T1 SGRE, T2 FSE, T2 FSE + FS	20	62 (44.9%)	41 (29.7%)	42 (30.4%)	62	1	0	75	100	98.7
A Singh^43^ ^e^	T2 SSFSE + FS, T2 FSE + FS, STIR, pre-GAD T1, post-GAD T1	<25	12 (30.0%)	36 (90.0%)	37 (92.5%)	12	1	0	27	100	96.4
N Nitta^44^	T1 SE, T2 FSE, T2 + FS	20	29 (78.4%)	1 (2.7%)	8 (21.6%)	29	1	0	7	100	87.5
L Incesu^46^	T1 SE, T1 CE FS, T2 FS FSE	25	34 (56.7%)	9 (15.0%)	11 (18.3%)	33	2	1	24	97.1	92.3

*ssEPI*, single-shot echo-planar imaging; *bSSFP*, balanced steady-state free precession; *RARE*, rapid acquisition with relaxation enhancement; *SPAIR*, spectral selection attenuated inversion recovery; *SENSE*, sequence, sensitivity encoding; *BTFE*, balanced turbo field echo; *SPIR*, spectral pre-saturation and inversion recovery; *SS-FSE*, single shot fast spin echo; *FSE*, fast spin echo; *STIR*, short tau inversion recovery; *SE*, spin echo; *DWI*, diffusion-weighted imaging; *True-FISP*, T2-weighted true-fast imaging with steady-state precession; *TIRM*, turbo inversion-recovery in magnitude; *TSE*, turbo spin echo; *HF*, half Fourier; *GRE*, gradient echo; *FLASH*, fast low-angle shot; *FS*, fat-suppressed; *HASTE*, half-Fourier acquisition single-shot turbo spin echo; *GAD*, gadolinium

^a^The values for sensitivity and specificity were calculated based on the TP, FP, FN, and TN, and are different to the values reported by the study that were calculated with a restriction to the 90 operative patients

^b^Results restricted to adults aged over 18

^c^This study had nine patients with equivocal MRI results, which were excluded from this table

^d^The values of sensitivity and specificity for this study are based on MRI interpretations by a surgeon who had 3 years of experience with abdominal MRI

^e^Results restricted to patients clinically suspected of appendicitis

Six secondary studies were identified^26,45,47–50^ and all but one^50^ examined the diagnostic accuracy of MRI in appendicitis—the results of these five secondary studies are summarised in Table 3. The largest of these was the 2021 Cochrane meta-analysis on the diagnostic accuracy of MRI for appendicitis, which calculated the pooled sensitivity (96%; 95% CI: 93–97%) and specificity (93%; 95% CI: 80–98%) for the subgroup of the general non-pregnant adult population.^26^

**Table 3:** Results of meta-analyses on the diagnostic accuracy of MRI for appendicitis

Author name	Year	Number of total patients	Sensitivity (%)	Specificity (%)
N D'Souza^26^	2021	7,492 (overall)	95 (95% CI: 94–97)	96 (95% CI: 95–97)
		1,088 (non-pregnant adults)	96 (95% CI: 93–97)	93 (95% CI: 80–98)
		2,794 (paediatric patients)	96 (95% CI: 95–97)	96 (95% CI: 92–98)
		2,282 (pregnant patients)	96 (95% CI: 88–99)	97 (95% CI: 95–98)
K Eng^45^	2018	287	89.9 (95% CI: 84.8–93.5)	93.6 (95% CI: 90.9–95.5)
M Repplinger^47^	2016	838	96.6 (95% CI: 92.3–98.5)	95.9 (95% CI: 89.4–98.4)
E Duke^48^	2016	2,665 ^a^	96 (95% CI: 95–97)	96 (95% CI: 95–97)
R Barger^49^	2010	363	97 (95% CI: 92–99)	97 (CI: 94–99)

^a^Results combining all paediatric, pregnant, and non-pregnant adults

We identified 12 studies that reported on topics unrelated to diagnostic accuracy.^5,50–60^ These studies are summarised in Table 4: three reported on trends of MRI use,^51,53,55^ two were reports about visualisation of the normal appendix,^58,59^ two were analyses of financial implications,^5,60^ one was a secondary study comparing simple versus perforated appendicitis using different imaging modalities,^50^ one reported on MRI to assess treatment response in non-operatively managed appendicitis,^52^ one was a decision analysis,^56^ one was a radiologist training report,^57^ and one was a survey of imaging prioritisation.^54^

**Table 4:** Summary of the studies identified in the systematic review that investigated topics other than the diagnostic accuracy of MRI

Author	Year	Topic	Finding
W Bom^50^	2020	The diagnostic accuracy of different imaging modalities for discriminating complicated from simple appendicitis ^a^	The available evidence was not sufficient for conducting a meta-analysis
A Agathis^51^	2019	The trend of MRI use according to the American College of Surgeons database	Of the 11,841 patients that received an appendicectomy in 2016, only 36 adult patients had received MRI
O Ozdemir^52^	2018	The utility of MRI in follow-up assessment	MRI was useful in the follow-up assessment of simple appendicitis cases after initial medical management
N D'Souza^5^	2018	Financial implications of imaging use for suspected appendicitis	The reduction in healthcare cost due to routine imaging was 68% less when using MRI instead of CT
V Tan^53^	2017	The trends of MRI use at 16 Canadian centres	MRI was generally not used for assessing appendicitis in non-pregnant adults in Canada, even at centres having MRI available 24 hours a day
M Agapova^54^	2017	Imaging preferences of U.S. physicians	Radiologists generally preferred contrast-enhanced CT over MRI to assess suspected appendicitis, whilst ED physicians had minimal preference in choosing between contrast-enhanced CT and MRI
M Repplinger^55^	2016	The trend of MRI use at one U.S. academic centre	MRI was never used for evaluating adults suspected of appendicitis between 1992 and 2014
S Kiatpongsan^56^	2014	Decision analysis on the benefits of MRI use	The advantage of MRI in minimising radiation exposure is substantial only if MRI demonstrates a minimum sensitivity of 91% when specificity is 100%, or minimum specificity of 62% when sensitivity is 100%
M Leeuwenburgh^57^	2012	Training of radiologists for interpreting MRI	Training of radiologists can improve the diagnostic accuracy of MRI for appendicitis
J Horowitz^58^	2011	Visualising normal appendixes	T2-weighted MRI could visualise 80% of normal appendixes
P Nikolaidis^59^	2006	Visualising normal appendixes	The reliability of MRI in visualising normal appendixes was variable
M Beinfield^60^	2005	Costs of MRI use	The costs of MRI varied significantly between 1996 and 2002

^a^This was a secondary study that investigated MRI, CT, and ultrasound. However, due to the insufficient evidence regarding MRI and ultrasound, meta-analysis was conducted only for CT

Three primary cohort studies were identified that reported on the diagnostic accuracy of MRI in identifying perforated appendicitis, reporting a sensitivity of 57–100% and a specificity 86–100%, respectively.^38,46,61^ The PPV and NPV of MRI features for discriminating complicated from simple appendicitis are shown in Table 5.

**Table 5:** PPV and NPV of individual MRI features for discriminating complicated from simple appendicitis, as identified in the systematic review; for the data reported by Leeuwenburgh et al.,^61^ the PPV and NPV were calculated to exclude cases without appendicitis, based on the reported values; the data reported by Zhu et al.^39^ was excluded due to an unusually high prevalence of complicated appendicitis

MRI feature	PPV	NPV
Appendiceal diameter > 7 mm^61^	26.3 (30/114)	100 (2/2)
Periappendiceal fat infiltration^61^	26.4 (29/110)	83.3 (5/6)
Periappendiceal fluid^61^	30.7 (27/88)	89.3 (25/28)
Absence of intraluminal air^61^	24.5 (26/106)	60 (6/10)
Appendicolith^61^	30.8 (16/52)	78.1 (50/64)
Extraluminal appendicolith^32^	100 (1/1)	73.5 (25/34)
Appendiceal wall defect^32^	75~85.7 (6/8 ~ 6/7)	85.2~85.7 (23/27 ~ 24/28)
Appendiceal wall destruction^61^	51.9 (14/27)	82.0 (73/89)
Phlegmon^32^	40.9~42.9 (9/22 ~ 9/21)	92.3~92.9 (12/13 ~ 13/14)
Abscess^32,61^	55.6 (10/18), 85.7~100 (6/7 ~ 6/6)	79.8 (79/99), 85.7~86.2 (24/28 ~ 25/29)
Extraluminal free air^32,61^	100 (1/1), 100 (2/2)	75 (87/116), 75.8 (25/33)
Restricted diffusion of appendiceal wall^61^	27.3 (27/99)	86.7 (13/15)
Restricted diffusion of appendiceal lumen^61^	27.9 (24/86)	82.1 (23/28)
Restricted diffusion of focal collections^61^	55.6 (15/27)	83.9 (73/87)

Eight primary cohort studies were identified that compared MRI and the reference standard with respect to their ability to identify alternative diagnoses when appendicitis was clinically suspected.^31,34,39,42,43,46,62,63^ The sensitivity and specificity of MRI for alternative diagnoses ranged between 77.0–100%^34,62^ and 94.9–100%,^34,43^ respectively. Seven of these studies additionally reported the data with a restriction to gynaecological pathologies, with the values of sensitivity and specificity ranging between 57.1–100%^34,63^ and 95.3–100%,^34,43^ respectively.

Four studies were identified that directly compared MRI with other assessment methods within homogenous populations. Repplinger et al. compared between MRI (sensitivity 96.9%, 95% CI: 88.2–99.5%; specificity 89.6%, 95% CI: 82.8–94.0%) and contrast-enhanced CT (sensitivity 98.4%, 95% CI: 90.5–99.9%; specificity 93.3%, 95% CI: 87.3–96.7%).^33^ Leeuwenburgh et al. compared between MRI (sensitivity 97%; specificity 93%) and ultrasound with selective use of contrast-enhanced CT (sensitivity 97%; specificity 91%).^37^ Incesu et al. compared between MRI (sensitivity 97%; specificity 92%) and ultrasound (sensitivity 76%; specificity 88%).^46^ Inci et al. compared between MRI (sensitivity 99%; specificity 100%) and the Alvarado scoring system (sensitivity 84.2%; specificity 66.7%).^64^

**Fig. 3:**
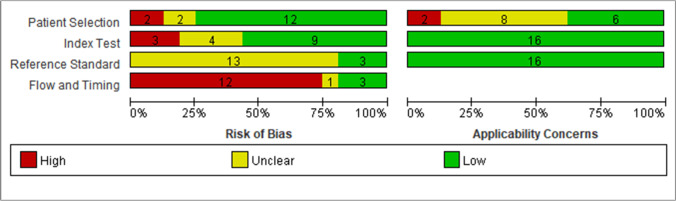
QUADAS-2 assessment findings for each domain represented as percentages

**Fig. 4:**
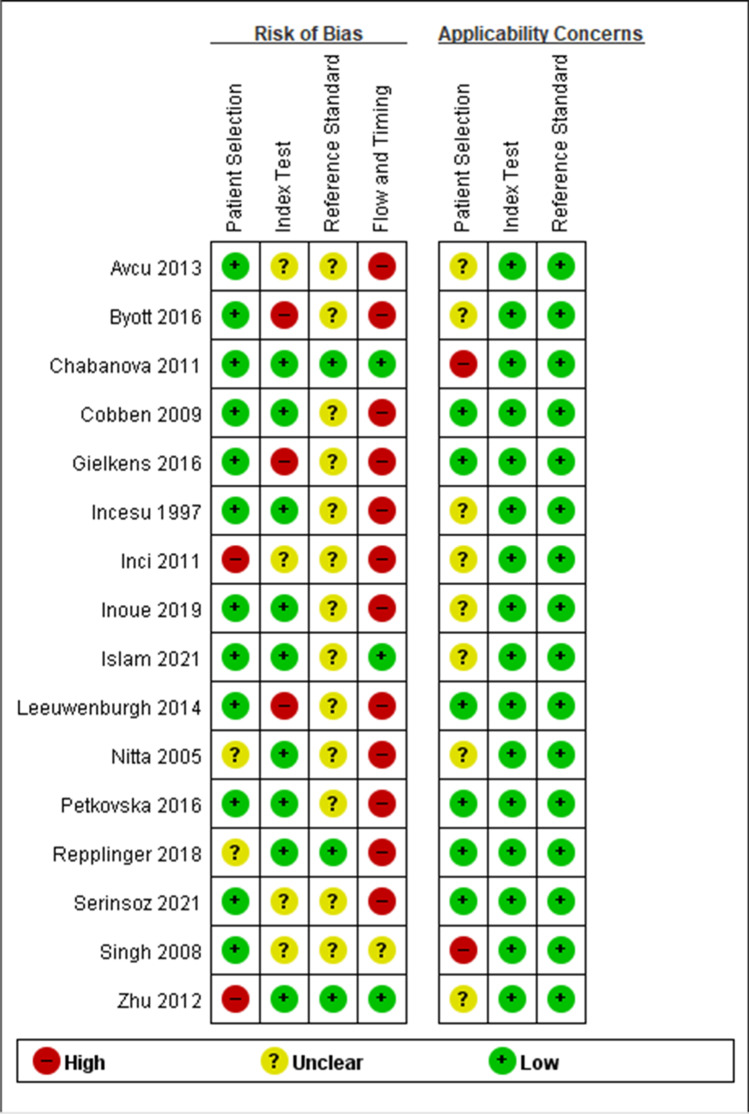
QUADAS-2 assessment findings for each domain of the diagnostic outcomes studies

Quality assessment results of the diagnostic accuracy studies are displayed in Figs. 3 and 4. The limitations of primary studies included insufficient descriptions of the reference standard, the employment of composite reference standards, and a significant portion of patients comprising paediatric, pregnant, or both subgroups.

## Discussion

In this study, we conducted a systematic review of the published literature concerning the use of MRI for evaluating possible appendicitis presentations in the general adult population. To our knowledge, this systematic review is the first to extend beyond the diagnostic accuracy of MRI and provides clinicians with an overview of the value of MRI for diagnosing appendicitis in the general adult population. Not only does MRI have high sensitivity and specificity for diagnosing appendicitis, but it also has a potential role in differentiating simple from complicated appendicitis and in identifying alternative diagnoses that are commonly seen when appendicitis is suspected, such as gynaecological diagnoses.

MRI has a promising diagnostic accuracy for acute appendicitis in the general adult population,^26^ with clinical parameters similar to CT (sensitivity 95%; 95% CI: 93–96%; specificity 94%; 95% CI: 92–95%)^12^ and superior to ultrasound (sensitivity 69%; 95% CI: 59–78%; specificity 81%; 95% CI: 73–88%).^20^ Direct comparisons within homogeneous populations suggested that the diagnostic accuracy of MRI is comparable to contrast-enhanced CT^33^ or ultrasound with selective use of contrast-enhanced CT,^37^ and superior to ultrasound^46^ or the Alvarado scoring system.^64^ No statistically significant difference exists when comparing MRI with CT or ultrasound for the visualisation of a normal appendix,^65^ likely due to the paucity of data published.

Five meta-analyses have been published on the diagnostic accuracy of MRI in appendicitis^26,45,47–49^ with the largest being the 2021 Cochrane Review.^26^ This study reported the pooled sensitivity and specificity of abdominal MRI separately in three distinct population groups: the pregnant, the paediatric, and the non-pregnant adult population.^26^ The headline overall (non-subgrouped) results reported on a population that is unrepresentative of the typical population presenting with appendicitis, with large numbers of paediatric patients (2,794), pregnant patients (2,282), and an uncategorised group of mixed patients (1,298). These patient groups combined to far exceed the size of the non-pregnant adult population (1,088 patients), though the diagnostic accuracy of MRI was similar across all subgroups. However, the Cochrane review was limited to the issue of diagnostic accuracy, whereas our paper also aims to cover the other knowledge domains regarding MRI in appendicitis.

The discrimination between simple and complicated appendicitis has significant ramifications on management, as surgeons can choose to medically manage simple appendicitis safely with antibiotics alone.^66^ Our results show conflicting values of sensitivity and specificity of MRI for identifying complicated appendicitis. This possibly reflects the variations in MRI interpretation criteria used in each study, as certain individual MRI features demonstrated a favorable ability to identify or exclude complicated appendicitis.^32,39,44^ Still, little evidence currently exists to support the use of MRI to identify perforated appendicitis, as also noted by a 2018 systematic review.^50^ The sensitivity and specificity of CT for identifying complicated appendicitis range between 28~95% and 71~100%, respectively.^50^

Our results suggest that the sensitivity of MRI for proposing alternative diagnoses is variable, despite the high specificity of MRI. The range of the reported values of sensitivity was greater when the alternative diagnoses were restricted to gynaecological pathologies. Using MRI to investigate gynaecological pathologies illustrated a significant advantage over CT, given the underwhelming performance of CT for the initial evaluation of adnexal pathologies.^67^ The variable results on the sensitivity of MRI may be partially attributable to the varying levels of MRI experience of the interpreting radiologists in different studies,^34,63^ as the sensitivity of MRI interpretation can improve with training.^62^

The amount of time needed to perform imaging has an obvious impact on the time it takes to diagnose and treat appendicitis. All but one of our studies reported an MRI duration of 25 minutes or less, with half of them reporting 15 minutes or less. Three studies used MRI protocols lasting four minutes or less, demonstrating that MRI for appendicitis can be performed rapidly. Preparation for a typical non-contrast MRI is minimal,^68^ whereas preparing a patient for CT (typically contrast-enhanced) often takes at least 30 to 60 minutes in clinical practice,^69^ for reasons such as intravenous cannulation for radiocontrast injection and administration and progression of oral contrast (commonly 1.5 to 2 hours).^68,70^ The use of contrast agents is common in most CT practices, with at least one contrast agent used over 80% of the time.^71,72^ By comparison, the MRI protocols used by the primary studies in our systematic review all used non-contrast sequences, with only one study adding a gadolinium contrast sequence, suggesting that contrast use is unnecessary in MRI of the appendix. The literature suggests that MRI is not significantly slower than CT in daily clinical practice, and indeed may be quicker in some scenarios, as was demonstrated with paediatric patients.^73^ Also, the use of MRI can avoid the need for contrast agents, which are well-recognised as a source of patient morbidity.^16,17^

Decision analysis shows substantial long-term patient benefit of receiving MRI over CT^56^ by virtue of avoiding the adverse effects of ionising radiation.^26^ Routine use of imaging including MRI significantly lowers healthcare costs by reducing the negative appendicectomy rate,^42^ though this cost reduction is 68% less when using MRI instead of CT.^5^ The improved identification of simple (versus complicated) appendicitis using MRI^38,46^ may allow risk stratification and more effective use of non-operative management, thus hypothetically reducing hospital costs, although this has yet to be formally studied. Similarly, MRI detection of gynaecological pathologies avoids the need to use both CT and ultrasound to identify these common differential diagnoses, making MRI potentially less costly in females. However, the exact financial implication of using MRI to assess suspected appendicitis is unclear due to the lack of a direct cost-benefit analysis.

Despite these advantages, an extremely small proportion of non-pregnant adult patients with suspected appendicitis receive MRI imaging in the USA and Canada,^51,53,55^ even at institutions that have MRI available 24 hours a day.^53^

Several limitations underlie the published meta-analyses, including the 2021 Cochrane Review. Firstly, the results of these meta-analyses generally do not extend beyond investigating sensitivity, specificity, and heterogeneity. Secondly, the meta-analyses published to date are subject to bias due to the lack of RCTs. A lack of RCTs introduces bias through unmitigated inter-cohort variability and the lack of randomisation or blinding. Thirdly, the generally poor reporting standards of the primary studies compromise the validity of the meta-analyses, as described by the 2021 Cochrane Review.^26^ Nevertheless, it is arguable that RCTs are unlikely to be funded or conducted given the currently available evidence.

Our systematic review contains multiple limitations. Heterogeneity of patient characteristics and study designs exists between individual primary studies. The presumed greater likelihood of publication of studies with positive conclusions about MRI would bring publication bias. Because the MRI experience of radiologists impacts diagnostic accuracy,^74^ our conclusions may not be generalised to centres where access to experienced MRI radiologists is limited.

Possible directions for future research may include cost-benefit analysis of MRI for appendicitis and the visualisation rate of the normal appendix given the lack of data in the current literature. More reports on the use of MRI to identify complicated appendicitis or propose alternative diagnoses would help better define the role of MRI in streamlining surgical decision-making in clinical practice.

## Conclusion

The diagnostic accuracy of MRI for appendicitis has already been shown to be excellent. Our systematic review identifies the evolving ability of MRI to help differentiate simple and complicated appendicitis or to identify alternative diagnoses, whilst avoiding the adverse effects of CT, such as the use of ionising radiation and the contrast agents. Whether MRI is less costly remains uncertain; however our systematic review demonstrates that MRI can be performed rapidly. Despite these benefits, MRI is rarely used in current clinical practice for the investigation of abdominal pain in adults, but our study suggests it has great potential to markedly benefit the pre-operative decision-making of surgeons treating appendicitis.
